# 国际肺癌研究会分期项目——采用外科治疗的非小细胞肺癌的预后因素和病理TNM分期

**DOI:** 10.3779/j.issn.1009-3419.2010.01.02

**Published:** 2010-01-20

**Authors:** Kari CHANSKY, Jean-Paul SCULIER, John J. CROWLEY, Dori GIROUX, Jan Van MEERBEECK, Peter GOLDSTRAW, on behalf of the International Staging Committee and Participating Institutions, 永波 杨, 志刚 李

**Affiliations:** 1 Statistics Department, Cancer Research And Biostatistics, Seattle, Washington; 2 Department of Intensive Care and Thoracic Oncology, InstitutJules Bordet, Université Libre de Bruxelles (ULB), Brussels, Belgium; 3 Department of Respiratory Medicine, University Hospital, Ghent, Belgium; 4 Royal Brompton Hospital, Imperial College, London, United Kingdom; 5 天津医科大学总医院，天津市肺癌研究所，天津市肺癌转移与肿瘤微环境重点实验室

**Keywords:** 非小细胞肺癌, TNM分期, 病理分期, 预后因素, 组织学, 细胞类型, IASLC肺癌分期项目

## Abstract

**目的:**

本研究的目的是在国际肺癌研究协会国际分期数据库中采用外科治疗的Ⅰ-ⅢA期非小细胞肺癌病例中，评价除肿瘤原发灶、病理淋巴结和转移（TNM）分期外，细胞类型、年龄和性别的影响。

**材料和方法:**

从提交至分期数据库的67 725例非小细胞肺癌（NSCLC）病例中，筛选出9 137例采用外科治疗的病例，这些病例的病理分期、年龄、性别和特殊组织细胞类型等信息均可获得。在亚组中分析记录行为状态和吸烟史。检验方法采用Cox比例风险回归和递归分割及合并（RPA）分析。

**结果:**

病理TNM分期、年龄以及性别均为生存的独立预后因素。尽管细支气管肺泡癌（BAC）亚型间存在潜在的异质性，其相对于其它细胞类型仍具有生存优势。修正比较提示罹患鳞癌相对于非BAC腺癌及大细胞癌具有微弱的生存优势，尽管此优势仅限于男性患者。RPA结果提示TNM分期为首要因素，年龄是各分期分组的预后因素。在RPA分析中未发现细胞类型具有预后价值。依据RPA的结果形成预后分组，诸分组的预后价值得到北美监视、流行病学、结局结果注册机构的认可。在资料可得的亚组中，行为状态和吸烟史均为预后因素。在回归模型中，吸烟状态的纳入未影响其它变量的效果。

**结论:**

年龄和性别已被证实为外科切除的非小细胞肺癌的重要预后因素。细胞类型的重要性次之，尽管归类于BAC的少部分病例相对于其它组织学类型具有生存优势，且鳞癌相对于非BAC腺癌具有微弱的生存优势。在未修正分析中，分期、年龄、性别和细胞类型间的不平衡可能会导致有关细胞类型的误导结果。在该分析中，病理TNM分类是最重要的预后因素。

在即将问世的国际抗癌联盟和美国癌症联合会制定的第7版恶性肿瘤的TNM分期中，国际肺癌研究会（ISALC）国际分期委员会已提交了关于修订肺癌的肿瘤、淋巴结和转移（TNM）描述^[1-4]^和分期分组^[5]^的提议。该提案是基于为此目的而综合多个数据库而成的大型数据库的数据而制定。作为研究中的一部分，国际分期委员会预后因素分会报告了除分期以外的其它预后因素对12 428例临床分期明确的非小细胞肺癌（NSCLC）病例生存的影响^[6]^。在修正疾病分期后，年龄、性别和行为状态均为生存的预后因素。在修正其它因素后，细胞类型仅在非小细胞肺癌类型中预测价值最小，而鳞癌细胞类型在整体上具有微弱的生存优势。然而，细胞类型似乎在Ⅲa期病例有重要意义。

在此，在来自IASLC数据库中病理分期明确的NSCLC采用外科治疗的9 137例患者中，我们主要检测了预后因素的一个亚组（细胞类型、年龄和性别）。相对于多个单一机构数据或基于群体登记资料的分析，大样本量病例数及组间治疗的相对同质性（外科手术可作为所有病例限定性治疗手段的一部分）使我们可以更详尽地探讨细胞类型的预后价值。关于腺癌和鳞癌组织学类型的相对预后，报道不一。考虑到患者分组中细胞类型的比例不同，我们应特别注意探讨分期、细胞类型、性别间与生存的关系。

## 方法

ISALC肺癌分期项目的方法学和主要计划已有报道^[2-5, 7]^。所有数据均为回顾性的，并经双方同意，以编码数据的形式转交癌症研究和生物统计学（CRAB），该编码数据不具有可识别的私有信息，并由数据提供方的适当监管许可进行。该项目已通过审查并被豁免CRAB机构审查委员会有关人类项目的进一步审查。

## 人群

总共有100 869例病例提交至国际数据库，在去除超出研究时间范围（1990-2000）、组织学类型不明、纳入时非新近诊断以及分期、治疗、随访资料不全的病例后，其中81 015例病例可用于分析。在可分析病例中，67 725例为非小细胞组织学类型。根据ISALC第7版TNM分期的提议，其中15 236例病理分期明确且外科治疗的病例具备足够的T、N、M描述资料可以进行重新分类。在这一分组中，9 137例Ⅰ-Ⅲa期病例来自细支气管肺泡癌（BAC）亚型数据库，与其它腺癌相区别，这些病例曾被当地病理医生判断为腺癌。这些病例的时间域大部分是在WHO 1999年颁布第3版肺癌分期指南之前^[8]^，因此许多被诊断为BAC的病例实际上是具有BAC组分的潜在腺癌，而不是无侵袭性的单纯BAC。尽管没有统一的细胞类型的组织病理学评价，而且我们也不能保证各组间细胞类型分配的一致性，尤其在鉴定BAC方面，但是将BAC或具有BAC特征的腺癌视为一单独类别是十分重要的。

所有病例的年龄、性别和细胞类型信息均可获得。2/3病例的行为状态信息不明；因此，这一因素未被列入初步分析中，而在一亚组中该因素得到探究。由于所有的病例均为手术备选者，在可进行行为状态评分的病例中经Zubrod评分后一般不超过1分。54%的病例吸烟史不详，因此亦被单独研究。而病历中记录有已接受过新辅助化疗的患者未被纳入分析范围。术后接受化疗的病例（约占资料可得病例的8.5%）可纳入分析。

纳入初步分析的病例来自于代表 18个国家的27个独立的数据库。贡献最大的是西班牙肺科与胸外科学会支气管肺癌协作组（GCCB-S，1 851例）和挪威登记处（1 737例），他们专门收集手术病例。大部分病例来自外科病例或医院联合会提交给中央登记处的手术病例。一小部分病例（143例）来自基于人群的登记处（收集采用所有治疗方法的患者），476例来自临床试验（表 1）。在纳入分析的9 137例病例中，1 950例病例亦被纳入先前有关临床分期病例的分析中。

**1:** 提交类型的地域特征

	总病例数	临床试验	医院联合会	登记病例^*a*^	外科病例
亚洲	1 135	0	0	0	1 135
澳大利亚	1 383	0	0	0	1 383
欧洲	4 818	10	1 851	1880	1 077
北美	1 801	466	0	0	1 335
所有地区	9 137	476	1 851	1 880	4 930
^*a*^分类包括了外科病例登记和基于人群的登记。 注：本表得到版权所有者© 2009 by the International Association for the Study of Lung Cancer复制许可					

## 统计学分析

将术后至任何原因导致死亡的时间定为生存期，中位生存期通过Kaplan-Meier方法计算。采用Cox回归分析评估预后组的总体生存期，运用的软件是9.0 PHREG windows版本的SAS系统。在回归分析中，以指标变量建立分期和组织学分类的分类模型。为了便于解释，设定年龄是分割点为70的二分法分类变量。尽管在以前的文献中这一组群的年龄的分割点为75，但现有的外科手术亚组中75岁以上病例所占比例较小。因此，选择70作为分割点，这与在“老年”患者的临床试验中经常引用的年龄分割点相一致。选择年龄变量二分法的决定已被回归模型结果验证：当采用年龄作为连续变量修正模型进行比较时，细胞类型、分期和性别的风险比均在±0.03以内。双变量（年龄和性别）的显著性检验采用Wald统计。对于每一个体假设，分期和组织学的个体水平比较也采用Wald统计。鉴于使用的变量数目和考虑的模型，统计学意义阈值修正为0.01。

通过分期（根据第7版TNM分期的提议）及关键预后因素：年龄、性别和细胞类型，运用递归分割及合并（RPA）分析^[9]^以产生树型结构模型。树形算法在包含可运于分析的9 137例病例的整体系统的训练集中展开，然后在美国国家癌症研究所的监视、流行病学、结局登记数据库（SEER）1998-2002时间域内的适合手术病例中检验其产生的预后分组的有效性。为了确保用于验证的各研究组间的可比性，仅属于适当的TNM分类且具有外科切除手术编码指征的非小细胞肺癌病例入选。SEER报道了最佳分期，在手术切除病例通常是指病理分期^[10]^。TNM分期分类来源于疾病编码及N分期（如肿瘤大小及其它肿瘤描述）的内容，并足可根据修订的分期标准进行重新分类。仅选取在提出的新分期中为Ⅰ-Ⅲa期的病例。

纳入RPA分析的变量包括分期（作为一个有序变量）、年龄（在单独的迭代次数中既可作为分类变量又可作为连续变量）、性别和细胞类型（作为指示变量的群）。应用RPA分析产生的有关生存数据的树形结构应用logrank检验，以选择数据的“最佳分割点”，进而形成最终分组及启动重新取样，以纠正分割算法的适应性本质。为了验证生存树状图结果的有效性，终末节点根据相似风险而分组且新形成的分组用SEER数据库进行评估。

## 结果

腺癌和鳞癌组织学类型占此项研究样本的大部分（分别占到36%和49%）。与腺癌（46%）相比，鳞癌在Ⅱ、Ⅲ期居多而在Ⅰ期较少（35%）。在性别与组织学方面存在不平衡。女性患者中，55%为腺癌，25%为鳞癌。与之相对，男性患者中，30%为腺癌，57%为鳞癌（表 2）。

**2:** 病理分期为Ⅰ-ⅢA期（根据IASLC提议的第7版TNM分期）数据库中以性别和分期分类的细胞类型分布，N=9 137

	细支气管肺泡癌	腺癌	鳞癌	大细胞癌	腺鳞癌	总计
女性						
Ⅰ期	183 (16%)	648 (56%)	247 (21%)	61 (5%)	10 (1%)	1 149
Ⅱ期	59 (8%)	363 (51%)	219 (31%)	64 (9%)	11 (2%)	716
Ⅲ期	34 (7%)	270 (57%)	117 (25%)	37 (8%)	18 (4%)	476
所有女性	276 (12%)	1 281 (55%)	583 (25%)	162 (7%)	39 (2%)	2 341
男性						
Ⅰ期	154 (6%)	892 (35%)	1 296 (51%)	175 (7%)	19 (1%)	2 536
Ⅱ期	59 (2%)	683 (27%)	1 572 (61%)	189 (7%)	58 (2%)	2 561
Ⅲ期	28 (2%)	479 (28%)	1 012 (60%)	124 (7%)	56 (3%)	1 699
所有男性	241 (4%)	2 054 (30%)	3 880 (57%)	488 (7%)	133 (2%)	6 796
女性+男性						
Ⅰ期	337 (9%)	1 540 (42%)	1 543 (42%)	236 (6%)	29 (1%)	3 685
Ⅱ期	118 (4%)	1 046 (32%)	1 791 (55%)	253 (8%)	69 (2%)	3 277
Ⅲ期	62 (3%)	749 (34%)	1 129 (52%)	161 (7%)	74 (3%)	2 175
所有患者	517 (6%)	3 335 (36%)	4 463 (49%)	650 (7%)	172 (2%)	9 137
注：本表得到版权所有者© 2009 by the International Association for the Study of Lung Cancer复制许可						

根据病理TNM分类（根据第7版的提议）将生存期进行排序，如预期（图 1A），中位生存期范围为Ⅲa期的19个月至ⅠA期的95个月。对于所有分期的细胞类型，BAC亚型的中位生存期为83个月，腺癌为45个月，鳞癌为44个月，大细胞癌为34个月，腺鳞癌为26个月（图 1B）。

**1:**
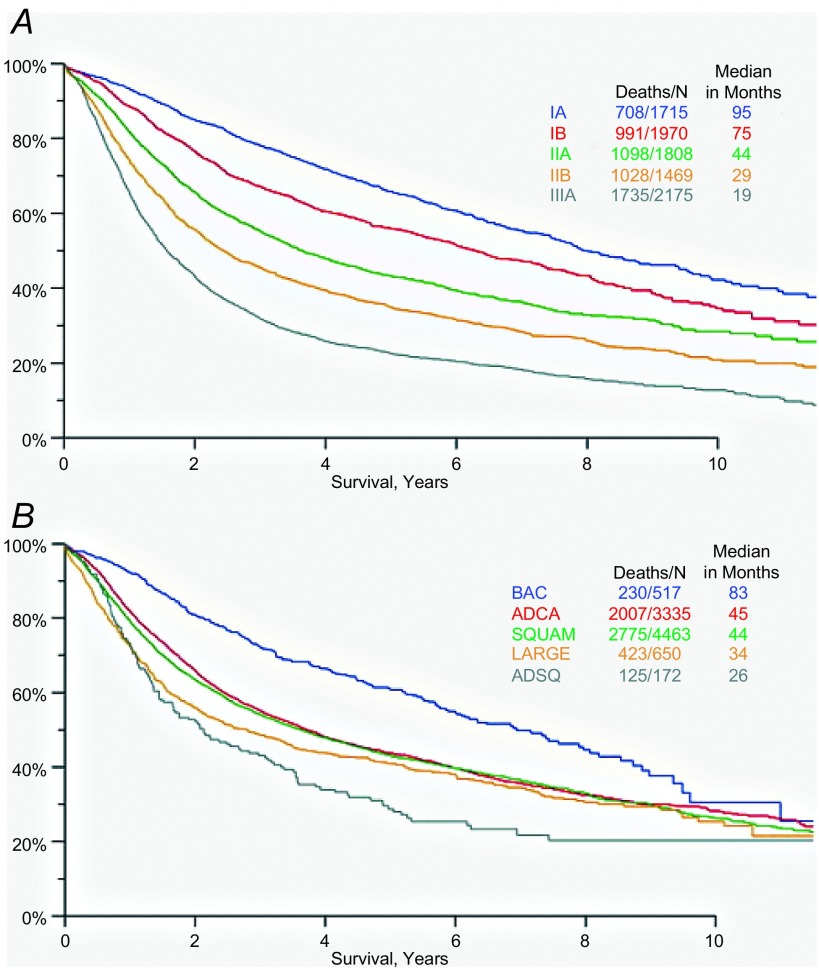
依照A：病理分期（IASLC提议的第7版）和B：细胞类型的生存曲线

对以下变量进行Cox比例风险回归分析：病理TNM分期（采用IASLC在第7版TNM分期中的建议）、年龄、性别以及组织学细胞类型（腺癌、鳞癌、大细胞癌、腺鳞癌和BAC）。未修正分析结果（每一因素均独立考虑）显示BAC和其它细胞类型之间、男性和女性患者之间、年龄≥70岁和 < 70岁患者之间存在显著性差异（表 3，未修正模型结果）。在所有分期和两种性别的未修正分析中，腺癌和鳞癌的预后无显著性差异。然而，在包括所有因素（细胞类型、病理分期、性别以及年龄）的模型中，与腺癌、大细胞癌相比，鳞癌具有显著的生存期优势，结果提示，在其它影响因素一致的情况下，鳞癌组织学类型具有较好的预后（表 3，修正模型结果）。大细胞癌和腺癌、腺鳞癌和其它非BAC组织学类型之间均无显著性差异。

**3:** 所有患者、男性患者及女性患者的单变量和多变量Cox比例风险比例回归模型的生存期统计和比较

因素	总体中位生存期（月）^*a*^/1年/5年所有患者	比较	非修正的H.R.^*b*^所有患者	修正后的H.R.^*c*^		
				所有患者	女性	男性
细胞类型						
BAC	83/92%/61%					
腺癌	45/82%/44%	腺癌*vs* BAC	1.56 (*p* < 0.000 1)	1.35 (*p* < 0.000 1)	1.42 (*p*=0.000 9)	1.25 (*p*=0.02)
鳞癌	44/79%/43%	鳞癌*vs*腺癌	1.03 (*p*=0.291)	0.86 (*p* < 0.000 1)	1.02 (*p*=0.80)	0.83 (*p* < 0.000 1)
大细胞癌	34/72%/41%	大细胞癌*vs*鳞癌	1.13 (*p*=0.046)	1.19 (*p*=0.000 9)	1.09 (*p*=0.45)	1.19 (*p*=0.003 2)
腺鳞癌	26/73%/29%	腺鳞癌*vs*大细胞癌	1.23 (*p*=0.046)	0.98 (*p*=0.846)	1.21 (*p*=0.38)	0.94 (*p*=0.60)
性别						
女性	66/85%/52%	男性*vs*女性	1.32 (*p* < 0.000 1)	1.21 (*p* < 0.000 1)	N/A	N/A
男性	40/79%/41%					
年龄						
< 70	49/81%/46%	年龄≥70 *vs* < 70	1.28 (*p* < 0.000 1)	1.51 (*p* < 0.000 1)	1.47 (*p* < 0.000 4)	1.52 (*p* < 0.001)
≥70						
TNM分期^*d*^	38/78%/38%					
ⅠA期	95/93%/66%					
ⅠB期	75/89%/56%	ⅠB *vs* ⅠA	1.33 (*p* < 0.000 1)	1.30 (*p* < 0.000 1)	1.39 (*p*=0.000 7)	1.25 (*p*=0.000 2)
ⅡA期	44/82%/43%	ⅡA *vs* ⅠB	1.39 (*p* < 0.000 1)	1.44 (*p* < 0.000 1)	1.48 (*p* < 0.000 1)	1.44 (*p* < 0.000 1)
ⅡB期	29/74%/35%	ⅡB *vs* ⅡA	1.28 (*p* < 0.000 1)	1.30 (*p* < 0.000 1)	1.43 (*p*=0.000 3)	1.27 (*p* < 0.000 1)
ⅢA期	19/65%/23%	ⅢA *vs* ⅡB	1.44 (*p* < 0.000 1)	1.46 (*p* < 0.000 1)	1.44 (*p* < 0.000 1)	1.46 (*p* < 0.000 1)
因素包括细胞类型、性别、年龄和病理分期（依照UICC/AJCC提议的第7版）。所有患者N=9 137；女性N=2 341；男性N=6 796。 ^*a*^中位总生存期来自Kaplan-Meier评估。 ^*b*^来自Cox比例风险回归分析的风险率和p值以组织学（以指标变量）、性别、年龄和分期（以指标）进行分别模拟。 ^*c*^来自多变量Cox比例风险回归模型的风险率和p值包括组织学、性别（以女性为参照）、年龄（< 70岁为参照）和分期（指标）。 ^*d*^提出的第7版TNM分期。 注：本表得到版权所有者© 2009 by the International Association for the Study of Lung Cancer复制许可						

组织学和性别间存在较小的但有统计学意义的相互作用（在对比完整和缩减模型的整体检验中*p*=0.006）。为了阐明该相互作用的本质，表 3分别显示了女性及男性患者的组织学、年龄和分期的生存期统计。在女性患者中，修正分期和其它因素后（尽管生存估计有利于腺癌）鳞癌与腺癌之间、其它非BAC组织学类型之间无显著性差异。然而，在男性患者中，相对腺癌和大细胞癌，鳞癌具有显著的生存优势，这或许是鳞癌在整体上具有较小生存优势的原因。

尽管危险比在Ⅱ、Ⅲ期分类中有利于鳞癌患者，且BAC只在Ⅰ期病例中（统计数据未显示）有显著的生存优势（*p*≤0.000 1），但在分期组别中，可达到显著性阈值0.01的任何非BAC组织学类型间并不存在差异。

分期、年龄、性别和细胞类型纳入RPA分析，以生成数据集的递归分割生存曲线。回顾经多重检测后有统计学差异的分割点，分期、年龄和（数据的有限部分）性别仍然是重要的变量（图 2）。与回归分析的结果不同，RPA方法得到的生存树可轻松解释在数据的特定亚组中不同因素的相对重要性。

**2:**
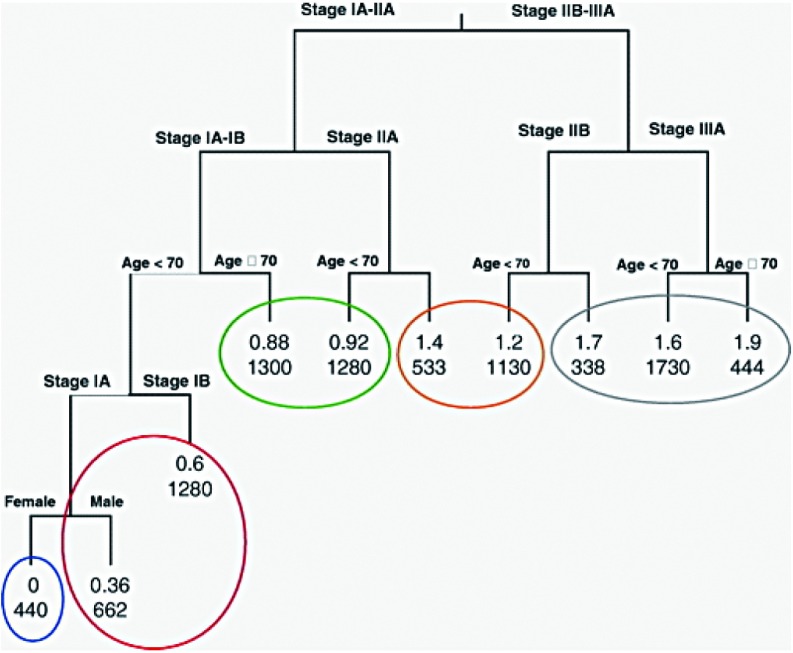
递归分割和合并分析得出的树状生存期结果

总体上最重要的因素是pTNM分期，且在分期分类中，年龄具有预后价值。除此之外，在70岁以下的ⅠA期分组中，女性是一个有利的预后因素。运用递归分割公式，在资料中的任一部分均未发现细胞类型为最重要的因素。对审计分割点的其它因素的相对重要性及地位而言，将年龄作为连续变量而不是分类变量的纳入不会改变树形图的结果，分类代表因此被确定（将70岁作为分割点）。RPA产生包括10个终端节点的生存树，根据风险比可将这10个终端节点分成具有相似预后的5个组别。根据如下标准来定义分组：

Ⅲa期-5组

ⅡB期-4组，如果年龄≥70岁则升高1级（至5组）

ⅡA期-3组，如果年龄≥70岁则升高1级（至4组）

ⅠB期-2组，如果年龄≥70岁则升高1级（至3组）

ⅠA期男性-2组，如果年龄≥70岁则升高1级（至3组）

ⅠA期女性-1组，如果年龄≥70岁则升高1级（至3组）

例如，80岁的ⅡA期患者应该划为4组。65岁的ⅠA期患者，如为男性应该划为2组，如为女性则为1组。

应用这些定义来验证SEER资料（n=9 221）可得到图 3中的生存曲线。所有毗邻分组在0.000 1水平均显著不同，毗邻分组的风险比分布于1.34至1.75之间。将细胞类型作为指示变量加入包含RPA分组变量的回归模型中是对整体检验的重要补充，这主要是因为鳞癌、腺癌和BAC之间存在差异。然而，值得注意的是这一补充仅将R^2^值（可被回归解释的变异百分比^[11]^）从21.8提高到22.6。

**3:**
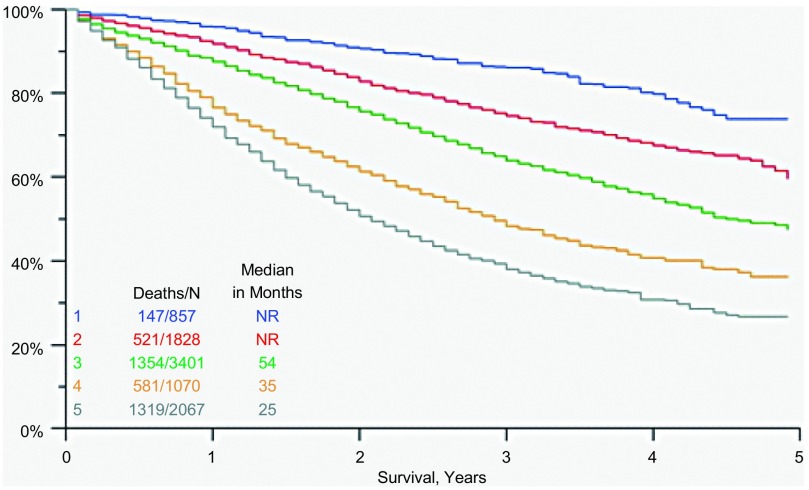
根据RPA、SEER资料的预后分组的验证，n=9 221

在有关行为状态资料可得的3 027例病例的修正性别、年龄和细胞类型后的分析中，行为状态对生存具有独立的预后价值。应用Zubrod评分，行为状态为1分的患者比为0分的患者预后差（H.R=1.16, *p*=0.005），小部分（n=35）行为状态为2分或以上的患者比为1分的患者预后差（H.R=1.61）。重要的是，在数据亚组的分析中，不同细胞类型、性别、年龄和分期的结果与行为状态不包括在内的整个数据集的结果相同。提示这些因素不依赖行为状态评分。

研究者检测了可得资料足以区分现时吸烟者（n=1 258）、曾吸烟者（n=1 155）及从不吸烟者（n=54）亚组的吸烟史。吸烟状态依细胞类型而不同，因此1.3%的鳞癌病例、9.4%的腺癌病例以及20%的BAC病例为从不吸烟者。在此亚组中，曾吸烟者和从不吸烟者间不存在差别（H.R=1.16, *p*=0.16），但现时吸烟者比曾吸烟者（H.R=1.21, *p* < 0.000 1）及从不吸烟者（H.R=1.41, *p*=0.001 7）预后差。尽管对“曾吸烟者”的定义可能有所不同，但可以肯定的是在单变量模型中吸烟意味着预后较差。在包括分期、细胞类型、性别和年龄的多变量分析中，尽管曾吸烟者和从不吸烟者间不存在差别（*p*=0.93），现时吸烟者和曾吸烟者间仍存在显著差别（*p*=0.001）。将吸烟状态作为一个因素加入模型中并没有影响其它因素观察到的效果。

## 讨论

以往对外科切除的非小细胞肺癌预后因素的分析已覆盖一些特定的范围，如：吸烟史^[12, 13]^、合并症^[14, 15]^、普通临床和人口统计学特征^[12, 16-22]^，病例数从少于100例到大约5 000例。有些研究，样本量从1 000例到19 000例不等，或许有重要的协变量研究，但它们不仅仅关注于外科治疗病例^[23-27]^。2002年的一项对文献的系统性综述发现，总体上研究的因素数目还比较少，结果具有异质性^[28]^。

基因表达^[11, 29-33]^研究是很有用的方法，特别是可以作为识别可从新辅助化疗中受益的早期患者（借助预后不良或预测性标记物）的一种手段。但是，这些研究往往涉及的患者数量比较少，而且这些患者一般来自不同的治疗模式。肺癌的基因表达模式研究（也有一些例外^[31-33]^）仅局限于细胞类型方面，新发现模式的预后功效与其它预后因素相结合的检测还比较少。基因标记只有在被反复证实可提供超出现有临床和解剖因素所提供的信息时，才可应用于临床。在此之前的肺癌研究中，我们主要依靠分期分类、一些新出现的生物标志物以及病人的临床特点。

现有分析使用的方法意在补充。递归分割分析（RPA）已用于确定预后分类，以期为分期系统的发展提供信息，例如，在肺癌（作为IASLC工作的一部分^[5]^）和多发性骨髓瘤中^[36]^。根据此类型的应用可以猜想，事实上患者将被划归为根据RPA结果而产生的分类中，以便评估预后。然而，基于树状图的模式，如RPA，经常被用作探索性分析的一部分，无须将最终分组用于临床实践即可被理解。例如，它们可被替代用于产生预后分类，以分析某治疗变量的效果^[37]^，或对现有分类方案提出改进^[38]^。RPA模型的分层结构可以阐明在传统回归模型中难以阐述清楚的各因素间的关系，有利于在患者的特定亚组中基于条件信息的关系的检测。以此项研究为背景，生存树状图为重要因素及其所处的重要亚层提供了图示。终端节点合并入分组便于对结果的验证，但在此情况下未设想将分组应用于临床实践。

在对来自IASLC肺癌分期项目中的采用外科治疗的非小细胞肺癌病例的分析中，年龄、分期分类及性别均对生存具有预后价值。相对于其它组织学类型，诊断为BAC的患者预后较好；而只有在修正了性别和分期的不平衡后，相对于腺癌和大细胞癌，鳞癌才有微弱的预后优势。而在女性患者中未发现相对于其它组织学类型鳞癌具有预后优势。

在具有完整信息的数据库内一个亚组中，与现时吸烟者相比，既往吸烟者预后较好，但是非吸烟者数目较少可能会妨碍我们得出有关此项分类的可靠结论。尽管修正了组织学类型或其它因素的一些报道忽略了吸烟状态的重要性^[15, 40]^，但多数研究报道认为非吸烟者和少吸烟者具有生存优势^[12, 13, 9]^。修正吸烟状态并没有改变细胞类型或其它因素的作用。该发现提示，即使吸烟史资料缺乏，基于性别、分期和细胞类型的结果仍然有效。然而，在该项和其它研究中非吸烟者大部分为腺癌分类（特别是BAC），因而吸烟和细胞类型并非完全独立，且两者均与性别相关。在NSCLC的研究中诸因素间的相互作用十分重要，吸烟状态的获取应添加到今后调查的数据收集过程中。随着更新数据收集很可能在不同分子标记物方面与实验室数据相联系，这一因素随之会变得日益重要。由于用作研究的非吸烟肺癌患者的数目较少，分析有时会采用吸烟强度（通过包/年或其它指标表示）^[12, 39]^而不是“曾经吸烟”对比“从不吸烟”的分类。然而，区分曾吸烟者与从不吸烟者依然很重要，且研究设计应纳入尽可能多的从不吸烟者。在数据可得的亚组中行为状态也具有预后价值，在外科病例的资料搜集中行为状态也是不容忽视的重要因素。

尽管人们几乎普遍认为性别和分期均为肺癌的预后因素，但有关组织学类型的影响报道不一，特别是有关两种最常见的非小细胞组织学类型的比较结果不一致。在此类分析中大部分的差异可用其它重要因素的遗漏来解释。

部分最新结果见表 4，该表显示了基于组织学类型的生存比较的不同结果。在可切除的NSCLC病例中，无法修正分期和性别是在腺癌细胞类型具有生存优势的研究的共同特征。例如，日本肺癌登记联合委员会发表的最新文献报道了2002年一年间在合作医院接受手术治疗的13 000余例肺癌病例。除报道女性患者具有生存优势的外，整体上腺癌细胞类型也具有较好的预后，其5年生存率为67%，而鳞癌仅为53%。作者认为预测因素的分析（包括组织学类型）并未修正其它因素，如分期或性别。这些修正可能导致不同的结果。

**4:** 由腺癌和鳞癌组成的非小细胞肺癌预后结果的新近研究

参考	人群	具有生存优势的细胞类型	研究特点
Asamura *et al*.^16^	日本肺癌登记联合会	腺癌	以pTNM分期为主。分期和性别均未修正。BAC未单独分类。
Foegle *et al*.^24^	下莱茵省，法国，区域登记	腺癌	修正至可切除*vs*其它。在可切除分类中未做进一步的分期修正。 BAC单独分类。
Caldarella *et al*.^25^	Tuscany注册人群	无区别	分期和性别均已修正。BAC单独分类但未进行单独分析（在非修正分析中腺癌在女性占优）。
OU *et al*.^22^	加利福利亚肿瘤登记ⅠA/ⅠB期病例	无区别	分期和性别均已修正。
Kawai *et al*.^12^	日本多医院登记的外科病例	无区别	仅ⅠA期，性别和吸烟状态已修正。（在未修正分析中非鳞癌占优）。
Ferguson *et al*.^20^	芝加哥大学外科病例	无区别	分期已修正（在未修正分析中腺癌占优）。
Berarda *et al*.^15^	Unicersitaà Politecnica del Marche Ⅰ-ⅢB外科病例	无区别	年龄、性别和吸烟状态均已修正。
Riquet *et al*.^17^	两个医院的外科病例	无区别	分期已修正。揭示了亚组内的变异。
Strand *et al*.^18^	挪威登记的外科亚群	鳞癌	分期和性别均已修正。BAC单独分类。此项分析中的一些患者亦被提交至ISALC数据库。
Wisnivesky *et al*.^19^	美国SEER数据库Ⅱ期外科病例	鳞癌	T、N分期以及性别均已修正，BAC的分类未见报道。
Pfannschmidt *et al*.^26^	海德堡胸科诊所外科病例	鳞癌	分期和性别均已修正。此项分析中的一些患者亦被提交至ISALC数据库。
Alexion *et al*.^21^	英国诺丁汉外科患者	鳞癌	分期和性别均已修正。
注：本表得到版权所有者© 2009 by the International Association for the Study of Lung Cancer复制许可			

作者还注意到BAC和含有BAC成分的腺癌未被区分，而均被纳入腺癌分类中。考虑到BAC亚分类在定义上已修改多次，且IASLC、ERS以及ATS即将联合为其提出新建议，这种分类也是合理的。在任何情况下，以任一标准定义的BAC似乎均预示着较好的预后^[8]^，因此将BAC归入其它腺癌将会提高腺癌分类整体上的生存预后^[17]^。相反，区别于其它腺癌而将BAC单独归为一种分类，可能会提示，相对于非BAC腺癌，鳞癌具有生存优势^[18]^。

修正了分期（包括或不包括性别）的研究发现，腺癌和鳞癌之间没有差别，或鳞癌有较好的预后。来自未修正分析及修正分析报道结果的研究发现，未修正分析更有利于腺癌，而修正分析未发现差别（表 4）。

在本研究中，分期与细胞类型、性别与细胞类型之间存在明显的不平衡。鳞癌病例中只有13%为女性，而腺癌中38%为女性。关于分期，46%的腺癌病例为Ⅰ期，而鳞癌中只有35%是Ⅰ期。虽然所有数据库均未来自明确的CT筛查项目，大多数的登记和联合会医院也未将这些病例排除在外。一些Ⅰ期的病例可能是筛查检测到的，腺癌病例的肿瘤体积倍增时间似乎更长^[43]^。而且，考虑到IASLC数据库是源于多中心的资料，一些BAC病例或具有BAC成分的腺癌病例在大的腺癌分类中仍未被识别出来，这些病例中大多数可能是早期的女性患者。修正分期和性别会弱化有利于腺癌细胞类型的任一不平衡的效果，并显示鳞癌细胞类型可能具有生存优势。在资料收集中，特别是在临床试验中，细胞类型仍将是重要的因素，因为新制剂的研究可依据组织学类型而表现出不同的效应^[44, 45]^。

利用IASLC国际分期项目数据库，我们可得出这样的结论：对于可采用外科治疗的病理分期为Ⅰ-Ⅲa期的NSCLC（根据IASLC提议的第7版TNM分期），除pTNM分期之外，年龄、性别和在较小的程度上的某些细胞类型均具有预后价值。分期仍然是最重要的因素，其次是年龄，在早期患者为性别。数据库中归类为BAC的病例不尽相同，从单纯非侵袭性BAC到含BAC组分的侵袭性腺癌。然而，这一分类与其它亚型的预后截然不同。对比两种最常见的非小细胞肺癌组织学类型，鳞癌较非BAC腺癌预后为好，尤其是在早期的男性患者中，然而，未被识别的BAC亚型归入腺癌是否会混淆鳞癌在男女性患者中的生存优势仍然存在争议。除其它因素外，在今后研究的数据采集中应收集吸烟史资料。

## 致谢

由AJCC基金“提高AJCC/UICC TNM肿瘤分期”资助。

礼来公司提供基金，并通过受限资助支持IASLC分期委员会建立数据库，并对第6版肺癌（分期）的TNM分期提出修改建议。礼来公司未参与委员会对资料的分析和对分期系统的修改建议。

## APPENDIX 1

### IASLC International Staging Committee

P. Goldstraw (Chairperson), Royal Brompton Hospital, Imperial College, London, UK; H. Asamura, National Cancer Centre Hospital, Tokyo, Japan; D. Ball, Peter MacCallum Cancer Centre, East Melbourne, Australia; V. Bolejack, Cancer Research and Biostatistics, Seattle, Washington, USA; E.Brambilla, Laboratoire de Pathologie Cellulaire, Grenoble Cedex, France; P. A. Bunn, University of Colorado Health Sciences, Denver, Colorado; D. Carney, Mater Misericordiae Hospital, Dublin, Ireland; K. Chansky, Cancer Research and Biostatistics, Seattle, Washington, USA; T. Le Chevalier (resigned), Institute Gustave Roussy, Villejuif, France; J. Crowley, Cancer Research And Biostatistics, Seattle, Washington, USA; R. Ginsberg (deceased), Memorial Sloan- Kettering Cancer Center, New York, USA; D. Giroux, Cancer Research And Biostatistics, Seattle, Washington, USA; P. Groome, Queen’s Cancer Research Institute, Kingston, Ontario, Canada; H. H. Hansen (retired), National University Hospital, Copenhagen, Denmark; P. Van Houtte, Institute Jules Bordet, Bruxelles, Belgium; J. -G. Im, Seoul National University Hospital, Seoul, South Korea; J. R. Jett, Mayo Clinic, Rochester, Minnesota, USA; H. Kato (retired), Tokyo Medical University, Tokyo Japan; C. Kennedy, University of Sydney, Sydney, Australia; H. Kondo, Shizuoka Cancer Centre, Sunto-gun, Japan; M. Krasnik, Gentofte Hospital, Copenhagen, Denmark; J. van Meerbeeck, University Hospital, Ghent, Belgium; T. Naruke, (deceased), Saiseikai Central Hospital, Tokyo, Japan; E. F. Patz, Duke University Medical Center, Durham, North Carolina, USA; P. E. Postmus, Vrije Universiteit Medical Center, Amsterdam, the Netherlands; R. Rami-Porta, Hospital Mutua de Terrassa, Terrassa, Spain; V. Rusch, Memorial Sloan-Kettering Cancer Center, New York, USA; N. Saijo, National Cancer Centre East, Kashiwashi, Japan; J. P. Sculier, Institute Jules Bordet, Bruxelles, Belgium; F. A. Shepherd, University of Toronto, Toronto, Ontario, Canada; Y. Shimosato (retired), National Cancer Centre, Tokyo, Japan; L. Sobin, Armed Forces Institute of Pathology, Washington, DC; W. Travis, Memorial Sloan-Kettering Cancer Center, New York, USA; M. Tsuboi, Tokyo Medical University, Tokyo, Japan; R. Tsuchiya (retired), National Cancer Centre, Tokyo, Japan; E. Vallieres, Swedish Cancer Institute, Seattle, Washington, USA; J. Vansteenkiste, Leuven Lung Cancer Group, Belgium; Yoh Watanabe (deceased), Kanazawa Medical University, Uchinada, Japan; and H. Yokomise (retired), Kagawa University, Kagawa, Japan.

### Participating Institutions

O. Visser, Amsterdam Cancer Registry, Amsterdam, The Netherlands; R. Tsuchiya and T. Naruke (deceased), Japanese Joint Committee of Lung Cancer Registry; J. P. Van Meerbeeck, Flemish Lung Cancer Registry-VRGT, Brussels, Belgium; H. _u¨lzebruck, Thoraxklinik am Universitatsklinikum, Heidelberg, Germany; R. Allison and L. Tripcony, Queensland Radium Institute, Herston, Australia; X. Wang, D. Watson and J. Herndon, Cancer and Leukemia Group B (CALGB), USA; R. J. Stevens, Medical Research Council Clinical Trials Unit, London, England; A. Depierre, E. Quoix and Q. Tran, Intergroupe Francophone de Cancerologie Thoracique (IFCT), France; J. R. Jett and S. Mandrekar, North Central Cancer Treatment Group (NCCTG), USA; J. H. Schiller and R. J. Gray, Eastern Cooperative Oncology Group (ECOG), USA; J. L. Duque-Medina and A. Lopez-Encuentra, Bronchogenic Carcinoma Co-operative Group of the Spanish Society of Pneumology and Thoracic Surgery (GCCB-S), Spain; J. J. Crowley, Southwest Oncology Group (SWOG); J. J. Crowley and K. M. W. Pisters, Bimodality Lung Oncology Team (BLOT), USA; T. E. Strand, Cancer Registry of Norway; S. Swann and H. Choy, Radiation Therapy Oncology Group (RTOG), USA; R. Damhuis, Rotterdam Cancer Registry, The Netherlands; R. Komaki and P. K. Allen, MD Anderson Cancer Center-Radiation Therapy (MDACC-RT), Houston, Texas; J. P. Sculier and M. Paesmans, European Lung Cancer Working Party (ELCWP); Y. L. Wu, Guangdong Provincial People’s Hospital, Peoples Republic of China; M. Pesek and H. Krosnarova, Faculty Hospital Plzen, Czech Republic; T. Le Chevalier and A. Dunant, International Adjuvant Lung Cancer Trial (IALT), France; B. McCaughan and C. Kennedy, University of Sydney, Sydney, Australia; F. Shepherd and M. Whitehead, National Cancer Institute of Canada (NCIC); J. Jassem and W. Ryzman, Medical University of Gdansk, Poland; G. V. Scagliotti and P. Borasio, Universita’ Degli Studi di Torino, S Luigi Hospital, Orbassano, Italy; K. M. Fong and L. Passmore, Prince Charles Hospital, Brisbane, Australia; V. W. Rusch and B. J. Park, Memorial Sloan-Kettering Cancer Center, New York, USA; H. J. Baek, Korea Cancer Centre Hospital, Seoul, South Korea; R. P. Perng, Taiwan Lung Cancer Society, Taiwan; R. C. Yung, A. Gramatikova, John Hopkins University, USA; J. Vansteenkiste, Leuven Lung Cancer Group (LLCG), Belgium; C. Brambilla and M. Colonna, Grenoble University Hospital-Isere Cancer Registry, France; J. Hunt and A. Park, Western Hospital, Melbourne Australia; J. P. Sculier and T. Berghmans, Institute of Jules Bordet, Brussels, Belgium; A. K. Cangir, Ankara University School of Medicine, Ankara, Turkey; D. Subotic, Clinical Centre of Serbia, Belgrade, Serbia; R. Rosell and V. Aberola, Spanish Lung Cancer Group (SLCG), Spain; A. A. Vaporciyan and A. M. Correa, MD Anderson Cancer Center-Thoracic and Cardiovascular Surgery (MDACC-TCVS), Houston, Texas, USA; J. P. Pignon, T. Le Chevalier and R. Komaki, Institut Gustave Roussy (IGR), Paris, France; T. Orlowski, Institute of Lung Diseases, Warsaw, Poland; D. Ball and J. Matthews, Peter MacCallum Cancer Institute, East Melbourne, Australia; M. Tsao, Princess Margaret Hospital, Toronto, Ontario, Canada; S. Darwish, Policlinic of Perugia, Italy; H. I. Pass and T. Stevens, Karmanos Cancer Institute, Wayne State University, USA; G. Wright, St. Vincent’s Hospital, Victoria, Australia; C. Legrand and J. P. van Meerbeeck, European Organization for Research and Treatment of Cancer (EORTC), Brussels, Belgium.
